# Determination of Carrier Frequency of Actionable Pathogenic Variants in Autosomal Recessive Genetic Diseases in the Turkish Cypriot Population

**DOI:** 10.3390/genes14101967

**Published:** 2023-10-20

**Authors:** Aziz Suat Gunsel, Mahmut Cerkez Ergoren, Hatice Kemal, Haniyeh Rahbar Kafshboran, Levent Cerit, Ayla Turgay, Hamza Duygu

**Affiliations:** 1 Department of Cardiology, Faculty of Medicine, Near East University, Nicosia 99138, Cyprus; aziz.gunsel@med.neu.edu.tr (A.S.G.); hatice.kemal@neu.edu.tr (H.K.); drcerit@hotmail.com (L.C.); hamza.duygu@neu.edu.tr (H.D.); 2 Department of Medical Genetics, Faculty of Medicine, Near East University, Nicosia 99138, Cyprus; 20212562@std.neu.edu.tr; 3 Laboratory of Medical Genetics, Near East University Hospital, Near East University, Nicosia 99138, Cyprus; ayla.turgay@neu.edu.tr

**Keywords:** allele frequency, mendelian disease, Turkish Cypriot, whole-exome sequencing

## Abstract

Whole-exome DNA sequencing is a rich source of clinically useful information for specialists, patients, and their families, as well as elucidating the genetic basis of monogenic and complex diseases in clinical diagnosis. However, interpreting and reporting variants encompassing exome and genome sequence analysis outcome data are one of the greatest challenges of the genomic era. In this study, we aimed to investigate the frequency and allele frequency spectrum of single nucleotide variants accepted as recessive disease carrier status in Turkish Cypriot exomes. The same sequencing platform and data processing line were used for the analysis of data from 100 Turkish Cypriot whole-exome sequence analysis. Identified variants were classified according to ACMG guidelines, and pathogenic variants were confirmed in other databases such as ClinVar, HGMD, Varsome, etc. Pathogenic variants were detected in 68 genes out of 100 whole-exome sequence data. The carriage rate was the highest in the *CYP21A2* gene, causing 21-hydroxylase deficiency (14.70%), 11.76% in the *HBB* gene causing β-thalassemia, 10.29% in the *BTD* gene causing biotinidase deficiency, 8.82% in the *CFTR* gene causing cystic fibrosis, 8.82% in the *RBM8A* gene causing thrombocytopenia-absent radius syndrome, which is an ultra-rare disease, and 5.88% in the *GAA* gene causing glycogen storage disease II. The carriage of pathogenic variants in other genes causing the disease (*GJB2*, *PAH*, *GALC*, *CYP11B2*, *COL4A3*, *HBA1*, etc.) was determined as less than 5.00%. Also, the identified variations in the mentioned gene within the examined population were reported. The most prevalent mutation in North Cyprus was a missense variant (c.1360 C>T, p.Pro454Ser) detected in the *CYP21A2* gene (rs6445), and the most frequently seen variant in the *HBB* gene was c.93-21G>A (rs35004220). We investigated reported pathogenic variants by estimating the lower and upper limits of carrier and population frequencies for autosomal recessive diseases, for which exome sequencing may reveal additional medically relevant information. Determining the lower and upper limits of these frequencies will shed light on preventive medicine practices and governmental actions.

## 1. Introduction

High-throughput next-generation sequencing (NGS) technology, especially targeted sequencing techniques, was introduced as a cost-effective and high-throughput method for human genome studies and clinical practices [1]. It is one of the most widely used methods because of its ability to identify the variants in hotspot and non-hotspot regions of genes [2]. Therefore, the identification rate of disease causative variants was raised by using this method, which paved the way for making molecular diagnoses and more effective treatments for patients even with rare diseases [3]. In addition, it helps to provide expanded carrier screening in populations without the omission of rare diseases, which means testing individuals without apparent symptoms of a genetic disease but may carry a single variant allele within a gene or genes linked to a particular condition, mainly Mendelian disorders [4]. Carrier screening is important in the prevention of pregnancies, with the heightened risk of being impacted by hereditary genetic conditions and in identifying who possesses a genetic disorder with a late or variable onset [5]. Numerous Mendelian disorders demonstrate autosomal recessive inheritance patterns, and approximately 1875 identified protein-coding genes are associated with recessive diseases, but this count may encompass only around 20% of the estimated total, indicating that the majority of recessive diseases remain uncharted [6]. As carriers of recessive disorders mostly have no clinical manifestation and no suggestive family history for these diseases, most couples are uninformed about the risk of having an affected child [7]. However, if both partners carry a pathogenic variant of the same gene or two different pathogenic variants of that gene or the female partner carries a disease-causing variant on her X chromosome, the risk of having an affected fetus elevates [8]. So, possessing epidemiological data regarding numerous hereditary conditions and carrier testing facilitates the prevention of the incidence of diseases in future generations by enabling the estimation of the genetic risk score in particular ethnic groups even in the absence of affected cases [9]. Moreover, preconception carrier screening is cost-effective due to its role in preventing Mendelian diseases, especially rare ones [7]. Indeed, the fundamental goal of carrier screening is to detect carriers, offer them genetic counseling and details about reproductive risks to facilitate their reproductive decision, and provide them with potential choices for reproductive assistance and prenatal tests, which are becoming more advanced, accurate, and rapid day by day with the progress of genetic technologies [10]. Recently, advanced genetic tests like NGS methods with the capability of screening hundreds of genetic disorders simultaneously as a panel (selective sets of genes associated with particular diseases) or whole-exome sequencing (WES) (determining exomes of all genes and not limited to a group of genes) have facilitated expanded carrier screening [6]. Undoubtedly, WES, as a major NGS technology, has gained increasing prominence in clinical applications and various scientific investigations [5]. Meanwhile, due to its ability to provide a more comprehensive evaluation compared to targeted carrier screening tests by examining a large portion of identified protein-coding genes, by identifying causative variants of genetic disorders effectively, and finding previously unknown pathogenic genes for monogenic diseases, it has been widely utilized for carrier screening [10].

In NGS, as a multiplex technology, different segments of DNA are sequenced simultaneously, which causes billions of reads, and the mapping of these reads yields massive data [3]. For the sake of the accurate and sufficient management of these data, various bioinformatics software have been developed [2]. Despite the multiple guidelines and software, variant interpreting and defining a molecular diagnosis based on the sequencing data remain challenging [8]. One of the most useful factors in assessing a variant’s potential pathogenicity is the frequency of its alleles in the general population [4]. According to ACMG-AMP guidelines, high allele frequency (BA1 and BS1) and the presence of a variation in the controls (BS2) are the criteria that shift the classification toward being benign, but higher frequency among affected individuals (PS4) and being absent from the controls (PM2) are the two criteria which have an impact on a variant classification toward pathogenicity [11]. In the direction of determining allele frequency in different populations, various reference datasets such as 1000 Genomes, Exome Variant Server (ESP), Exome Aggregation Consortium (ExAC), and the Genome Aggregation Database (gnomAD) were created [12]. In spite of abundant samples in these projects, allele frequency and disease prevalence are not fully clear in many areas. Hence, a lot of race-matched control studies have taken place around the globe to fill this gap [1]. In this study, we aimed to estimate the prevalence of monogenic autosomal recessive diseases according to the frequency of pathogenic alleles in the northern part of Cyprus using the whole-exome sequencing method.

## 2. Material and Methods

### 2.1. Demographics of Studied Subjects

The study group contains 100 individuals (58 males and 42 females) who came to Near East Hospital due to the Myocardial bridge. Patients with known genetic syndromes like Down syndrome, Turner syndrome, Andersen–Tawil syndrome, Leopard syndrome, and 22q11 deletion syndrome were excluded from this study. Informed consent forms were taken from all participants, and the study protocol was in acquiescence with the Helsinki Declaration and approved by the institutional Ethics Committee (Approval number: YDU/2020/85-1210).

### 2.2. Genomic Analysis Workflow

Venous blood samples were obtained from 100 participants, and genomic DNA was isolated (EZ1 Advanced XL Blood, QIAGEN, Hilden, Germany) from dry blood spots in filter cards (CentoCard) containing ethylenediaminetetraacetic acid (EDTA) for whole-exome sequencing analysis, as well as copy number and mitochondrial DNA analysis following the manufacturer’s instructions (QIAamp DNA Blood Mini QIAcube Kit, Qiagen, Valencia, CA, USA). Extracted DNA samples were stored at −20 °C. Prior to the sequencing analysis, DNA quality and concentration for each individual were determined using a photometric spectrometer (OD260/OD280 1.8–2.0).

The service was procured from CENTOGENE^®^ (Rostock, Germany) for whole-exome sequencing analysis. DNA-captured probes were used for enzymatically digested and enriched target sites of genomic DNA. The target region (~<98% of GRCh37/hg19) covered approximately 41 Mb of the human coding region, flanking ±20 intronic nucleotides of genes and the mitochondrial genome. Sequencing of the generated library was performed on an Illumina platform to achieve at least 20× depth. The company used its own in-house bioinformatics pipeline. Variant calling, annotation, and extensive variant filtering were applied to the GRCh37/hg19 genome assembly, including read alignment and the revised Cambridge Reference Sequence (rCRS) of human mitochondrial DNA (NC_012920). Any variant with a minor allele frequency (MAF) less than 1% and registered as a disease-causing variant in other databases was reported. Variants were categorized according to ACMG guidelines (pathogenic; likely pathogenic; variant with unknown significance (VUS); likely benign; benign) [12]. A whole-exome sequencing (WES) analysis does not cover larger deletions/duplications involving intron–exon boundaries, re-amplification, or methylation abnormalities. The .vcf files can be given upon request to mahmutcerkez.ergoren@neu.edu.tr.

## 3. Results

A WES including next-generation sequencing (NGS)-based copy number variation (CNV) analysis was performed on 100 participants. The targeted nucleotide coverage was ≥20×, and it covered ~99.35% of the interesting regions. The patient group consisted of 42% females and 58% males. The ethnic origin distribution was 45.5% Turkish, 53.5% Turkish Cypriot, and 1% Turkoman (Table 1).

Firstly, variants with high allele frequencies in databases such as the 1000 Genome Project (1KGP) (2500 samples; http://www.1000genomes.org, accessed on 13 September 2022), the Exome Variant Server (ESP) (6500 WES samples; https://evs.gs.washington.edu/EVS/), and the Exome Aggregation Consortium (ExAC) database (61,468 multiethnic individuals) were filtered out. Additionally, we focused on any variant function (nonsense, frameshift, conserved splice site, and missense) that affects protein structure, with supporting evidence on the zygosity/segregation/functional importance of the gene. Gene selection was conducted based on OMIM^®^ phenotypes and variant databases; any variant associated with severe and early-onset disease and reported as “pathogenic” and “likely pathogenic” were determined and listed. Gene variants associated with late-onset diseases with unclear penetrance and/or cancer-related genes with onset in adulthood were not included. 

Table 2 showed that 100 participants had pathogenic and/or likely pathogenic variants relevant to Mendelian diseases. In total, 14.7% of the studied population were carriers for *CYP21A2* gene variants, which are associated with autosomal recessive congenital adrenal hyperplasia with 21-hydroxylase deficiency (OMIM^®^: 201910), 11.7% had *HBB* gene variants that are relevant to autosomal recessive β-thalassemia (OMIM^®^: 613985), which is the most common carrier pattern in Cyprus, 10.29% were found to carry *BTD* gene variants that cause biotinidase deficiency (OMIM^®^: 253260), 8.82% for *CFTR* gene variants that cause cystic fibrosis (OMIM^®^: 602421), 8.82% for the *RBM8A* gene that causes thrombocytopenia-absent radius syndrome (OMIM^®^: 605313), 5.88% for the *GAA* gene that causes glycogen storage disease II (OMIM^®^: 232300), and 4.41% for *GJB2* and *PAH* genes that cause autosomal recessive deafness 1A (OMIM^®^: 220290) and Phenylketonuria (OMIM^®^: 261600), respectively. The carrier rate was detected as 2.94% for *ATP7B*, *GALC*, *PYGM*, *COL4A3*, *CYP11B2*, *ECHS1*, *HBA1*, *LAMA2*, *OPHN1*, and *POLR3A* genes, which cause Wilson disease (OMIM^®^: 277900), Krabbe disease (OMIM^®^: 245200), McArdle disease (OMIM^®^: 232600), Alport syndrome 2 (OMIM^®^: 203780), hypoaldosteronism (OMIM^®^: 203400 and 610600), mitochondrial short-chain enoyl-CoA hydratase 1 deficiency (OMIM^®^: 616277), α-thalassemia (OMIM^®^: 604131), muscular dystrophy (OMIM^®^: 607855 and 618138), and leukodystrophy or Wiedemann–Rautenstrauch syndrome (OMIM^®^: 607694 and 264090), respectively. The carrier rate for the *OPHN1* gene, which is responsible for X-linked syndromic intellectual developmental disorder (OMIM^®^: 300486), was estimated as 2.94%. X-linked *G6PD*-deficient (favism) hemolytic anemia (OMIM^®^: 611162), which is caused by the *G6PD* gene and many others listed in Table 2, was found at 1.47% in the studied population.

Table 3 demonstrates the observed mutations of each gene in the study population with details like nucleotide change, amino acid alteration, and SNP IDs. Among all 84 pathogenic and/or likely pathogenic variants that were encountered in the 64 found genes in this study, missense mutations have the highest frequency. As well frameshift, nonsense, splice site variants, and other mutations were noted. For some genes, only one variation was discovered in carriers, but for some others there was more than one. These mutations were as follows: five mutations in *CYP21A2* gene (c.850 A>G/p.Met284Val, c.844 G>T/p.Val282Leu, c.293-13 C>G, c.1174 G>A/p.Ala392Thr, and c.1360 C>T, p.Pro454Ser), three mutations in *HBB* gene (c.93-21 G>A, c.118 C>T/p.Gln40*, and c.20 A>T/p.Glu7Val), three mutations in *BTD* gene (c.1336 G>C/p.Asp446His, c.1489 C>T/p.Pro497Ser, and c.1330 G>C/p.Asp444His), six mutations in *CFTR* gene (c.3872 A>G/p.Gln1291Arg, c.2249 C>T/p.Pro750Leu, c.3205 G>A/p.Gly1069Arg, c.2991 G>C/p.Leu997Phe, c.3472 C>T/p.Arg1158*, and c.2421 A>G/p.Ile807Met), three mutations in *GAA* gene (c.1194+45 G>A, c.-32-13 T>G, and c.1828 G>A/p.Ala610Thr), two mutations in *GJB2* gene (c.35del/p.Gly12Valfs*2 and c.269 T<C/p.leu90Pro), two mutations in *PAH* gene (c.1066-11 G>A, c.1139 C>T/p.Thr380Met), two mutations in *ATP7B* gene (c.122 A>G/p.Asn41Ser, c.3053 C>T/p.Ala1018Val), two mutations in *GALC* gene (c.956 A>G/p.Tyr319Cys, c.1901 T>C/p.Leu634Ser), two mutations in *ECHS1* gene (c.476 A>G/p.Gln159Arg, c.538 A>G/p.Thr180Ala), two mutations in *POLR3A* gene (c.1771-7 C>G, c.1771-7 C>G), a mutation in *RBM8A* gene (c.-21 G>A), a mutation in *PYGM* gene (c.808C>T/p.Arg270*), a mutation in *COL4A3* gene (c.4421 T>C/p.Leu1474Pro), a mutation in *CYP11B2* gene (c.788T>A/p.Ile263Asn), a mutation in *HBA1* gene (c.95+2_95+6 del), a mutation in *LAMA2* gene (c.2451-2 A>G), a mutation in *OPHN1* gene (c.1484del/p.Lys495Argfs*9), a mutation in *MYH7* gene (c.2609 G>A/p.Arg870His), a mutation in *IFIH1* gene (c.2465 G>A/p.Arg822Gln), a mutation in *EXPH5* gene (c.2004_2014del, p.(Thr670Cysfs*8), a mutation in *POMT1* gene (c.598 G>C/p.Ala200Pro), a mutation in *ACADM* (c.244 dup/p.Trp82Leufs*15), a mutation in *NAGS* gene (c.1552 G>A/p.Ala518Thr), a mutation in *SUCLG1* gene (c.823 dup/p.Ser275Phefs*38), a mutation in *ASS1* gene (c.116 8G>A/p.Gly390Arg), a mutation in *CYP27A1* gene (c.1184+1 G>A), a mutation in *AMT* gene (c.992 G>A, p.Arg331Gln), a mutation in *PCCA* gene (c.1495 del/p.Ile499Serfs*19), a mutation in *SEC23B* gene (c.40 C>T/p.Arg14Trp), a mutation in *FBXL4* gene (c.616 C>T/p.Arg206*), a mutation in *CTSK* gene (c.721 C>T/p.Arg241*), a mutation in *C12orf65* gene (c.248 del/p.Val83Glyfs*2), a mutation in *CCBE1* gene (c.521 G>A, p.Cys174Tyr), a mutation in *CDK10* gene (c.609-1 G>A), a mutation in *CEP135* gene (c.2722 C>T/p.Arg908*), a mutation in *CHRNG* gene (c.753_754 del/p.Val253Alafs*44), a mutation in *MUTYH* gene (c.884 C>T/p.Pro295Leu), a mutation in *CLCN1* gene (c.854 G>A/p.Gly285Glu), a mutation in *COL18A1* gene (c.4768_4769 del/p.Leu1590Valfs*72), a mutation in *KIAA0586* gene (c.428 del/p.Arg143Lysfs*4), a mutation in *SLC25A26* gene (c.316 C>T/p.Arg106*), a mutation in *FANCE* gene (c.355 C>T/p.Gln119*), a mutation in *FTCD* gene (c.990 dup/p.Pro331Alafs*2), a mutation in *G6PD* gene (c.653 C>T/p.Ser218Phe), a mutation in *GHR* gene (c.739 T>C/p.Tyr247His), a mutation in *HBA2* gene (c.*92 A>G), a mutation in *MFSD8* gene (c.1361 T>C/p.Met454Thr), a mutation in *MOCS2* gene (c.471_477delinsG/p.Leu158_Lys159 del), a mutation in *PGAP3* gene (c.*559_*560inv), a mutation in *NPHS1* gene (c.1379 G>A/p.Arg460Gln), a mutation in *PKLR* gene (c.1456 C>T/p.Arg486Trp), a mutation in *PYROXD1* gene (c.464 A>G/p.Asn155Ser), a mutation in *TNFRSF13B* gene (c.204 dup/p.Leu69Thrfs*12), a mutation in *ABCD1* gene (c.1699 C>T/p.Gln567*), a mutation in *NPC1* gene (c.506 A>T/p.Asn169Ile), a mutation in *PLOD2* gene (c.1856 G>A/p.Arg619His), a mutation in *DHTKD1* gene (c.2185 G>A/p.Gly729Arg), a mutation in *GALT* gene (c.940 A>G/p.N314D), a mutation in *SBDS* gene (c.258+2 T>C), a mutation in *WDR35* gene (c.1922 T>G/p.Leu641*), a mutation in *ABCC6* gene (c.3421 C>T/p.(Arg1141*), a mutation in *COLQ* gene (c.1082 del/p.Pro361Leufs*65), and a mutation in *GCH1* gene (c.671 A>G/p.Lys224Arg).

## 4. Discussion

Genetic disorders as the main reason for infant mortality in the United States are responsible for about one-fifth of infant fatalities that occur each year. The advancement in genomic technology has facilitated cost-effective, comprehensive genetic screening across different ethnic groups, which allows for preconception and prenatal screening for the prevention of more than 100 genetic conditions with recessive inheritance patterns [5,10]. In the current study, we aimed to investigate the frequency and allele frequency spectrum of single nucleotide variants accepted as recessive disease carrier status in Northern Cyprus using the whole-exome sequencing method. The use of the WES test will enhance the rate of detection for numerous disorders and gene variations. WES raw data were analyzed, and all pathogenic and likely pathogenic variants associated with the severe and early-onset disease were classified. If any of the participants provide consent to know, information and genetic counseling will be administered to them and their families about the identified variants that indicate additional genetic risks or diagnoses. Cases were carriers of most (14.7%) of these variants within the *CYP21A2* gene that is associated with autosomal recessive congenital adrenal hyperplasia with 21-hydroxylase deficiency. According to the information provided by Phedonos et al. in 2013 on the carrier frequency of *CYP21A2* mutations in Cyprus, 1 out of 25 to 1 out of 10 newborns were reported as a carrier of a mutation in this gene. Also, Baumgartner-Parzer and colleagues documented the carrier frequency of the *CYP21A2* gene in their 2005 study, in which they screened newborns, as 9.5% in the middle European population. Furthermore, Gialluisi et al. (2017) conducted a study in two distinct regions of Italy, in which they calculated the prevalence of a mutation in the CYP21A2 gene and found it to be high. Thus, our findings align with prior research, indicating a heightened prevalence of CYP21A2 gene variations in regions such as Cyprus, Italy, Middle European populations, and Turkey [13,14,15,16]. Typically, congenital adrenal hyperplasia’s severity corresponds to the combination of more or less severe mutations (homozygous or compound heterozygous) and, as a result, encompasses a broad spectrum of disease manifestations, which could hold significant implications for genetic and prenatal counseling [15,16]. Thus, screening the individuals for *CYP21A2* gene variants in this population should be recommended in the future. On the other hand, even though the individuals who are heterozygotes for CYP21A2 gene mutations do not exhibit clinical symptoms, they still possess a well-defined phenotype. Nordenström et al. in a study in 2019 investigated the mortality rate and cause of death between CYP21A2 mutation carriers and population controls and suggested that carriers might experience lower mortality while facing severe infections, with a possible emphasis on pneumonia. One theory proposes that an enhanced ability to produce cortisol hormone during acute circumstances could explain the potential evolutionary benefit of being a carrier of CYP21A2 mutation, which may have contributed as an effective factor in survival advantage and could explain the widespread prevalence of CYP21A2 carriers across the globe [17]. According to the fact that an inactive pseudogene (*CYP21A1P*) with 98% similarity to the functional *CYP21A2* gene in exonic sequences and 96% in introns exist within the major human histocompatibility complex (HLA), approximately 30 kb away from the *CYP21A2* gene, correct genotyping and distinguishing these genes using PCR-based methods are challenging. Because of high levels of homology, amplification occurs in both genes by most primers at the same time [18]. Although genotype–phenotype correlation analysis can help to recognize false positive cases and reduce misdiagnosis, it was impractical due to the nature of our research on carriers. So, segregation analysis and studying flanking microsatellites in family members can be helpful solutions to reduce this problem [16].

Not surprisingly, β-thalassemia carriers had the second most (11.76%) seen pathogenic variants. The findings manifest a lower frequency of *HBB* mutation than *CYP21A2* mutations in the Turkish Cypriot population rising in Northern Cyprus, which can be the result of the premarital screening program for Thalassemia that started in 1984. According to Bozkurt G, 2007, this project significantly shrank the incidence of Thalassemia between 1991 and 2001, and between 2002 and 2007, as no thalassemia babies have been born in Northern Cyprus since then [19]. Accordingly, having guidelines for the carrier screening of congenital adrenal hyperplasia and preconception and prenatal screening programs for *CYP21A2* mutations seems to be the requisite in Northern Cyprus considering the preventive medicine strategies on the island. Also, it seems reasonable to offer *CYP21A2* mutation screening to the families before gamete donation or adoption. Despite the high carrier frequency of Familial Mediterranean Fever in the Turkish population [20], no variants in the *MEFV* gene were observed in our study in Northern Cyprus. This may be the result of the founder effect in the Turkish Cypriot population, but more data are required to prove it.

Furthermore, in this study, the prevalent mutations of each gene were detected in carriers which can offer several benefits and are essential for personalized medicine, improving the overall health and wellbeing of individuals and communities. Gathering data on diverse gene mutations, single nucleotide polymorphisms, or copy number variations has expedited research by identifying target areas for genetic research, leading to the development of new therapies, drugs, and interventions. Likewise, it allows for better and cost-effective healthcare planning and the management of genetic conditions by focusing resources on the most prevalent genetic conditions and reducing unnecessary testing and treatments. This information supports the training and education of healthcare professionals and helps them to be more prepared to diagnose, treat, and manage individuals with these mutations, which can lead to the early detection of genetic disorders, timely interventions and treatments, suggesting proper prenatal tests, or considering alternative reproductive options. In addition, these data assist public health organizations in implementing preventive measures, such as screening programs or genetic counseling services, to reduce the burden of genetic diseases. Additionally, the outcomes of this study can aid individuals and families to make informed decisions about their health and genetic risks and reduce the stigma associated with genetic disorders, as it becomes a more widely understood aspect of a population’s genetic makeup.

Some restrictions in this study may lead to either underestimation or overestimation of our results, such as the probable underestimation of carrier numbers due to the small size of the population under examination, which may not accurately represent the entire population of Turkish Cypriotes, as well as the absence of whole-genome sequencing (WGS) data and lack of information on other mutations like promoter and intronic mutations. Another effective issue is the restriction of our estimations on recognized pathogenic or likely pathogenic variants, which leads to missing unidentified variations and underestimating the carrier frequency or overestimated carrier frequencies owing to the incorrect classification of some variants as being pathogenic or likely pathogenic, even though we adhered to the ACMG criteria for classifying all variants. Additionally, the incorrect classification of variants of uncertain significance (VUS) is a challenge that may persist until there is sufficient evidence linking these variants to the disease, which may result in the underestimation of carrier frequency due to the classification of certain potentially pathogenic variants as VUS as a consequence of their high frequency or the absence of supportive clinical data. Moreover, existing estimates may only capture a fraction of the total occurrence of recessive diseases, as statistical projections suggest that some of these conditions remain unidentified or undescribed.

Most of the carrier statuses were relevant to autosomal recessive monogenic disorders; therefore, genetic counseling would be beneficial for their family. However, the *COL4A3* gene variants were also associated with autosomal dominant disorders; thus, patients should be re-examined for their possible clinical phenotype considering the penetrance degree in autosomal dominant disorders. However, these variants may help to close a potential diagnostic gap regarding the current clinical picture, but this ethnicity-based information should be used with caution because of challenges like individuals of mixed ethnicity, adoptive backgrounds, or unknown ancestral heritage. This information may be used in the further differential diagnosis processes and orthogonal validation of relevant variants and has the advantage of easing the distress experienced by families during the initial phase of diagnosis and the advantage of informing relatives about their elevated genetic risks. Couples facing genetic risks can explore alternative methods for beginning a family while aligning with their religious and ethical beliefs. This study also is the first WES study performed in the Turkish Cypriot population; therefore, the results, especially for autosomal recessive diseases and their carrier status, have a public health advantage in terms of being significant to shed light on preventive medicine practices and aiding in re-shaping public health and government policies to reducing the expenses associated with the initial evaluation of a newborn with a rare disease of unknown cause and preventing the significant healthcare expenses incurred by society due to patient care.

## Figures and Tables

**Table 1:** General demographics of the studied group.

Gender	Age	Ethnic Origin
M 39 (58.2%)	M 62.21 ± 12.02	Turkish (45.5%)
F 28 (41.8%)	F 58.02 ± 10.49	Turkish Cypriot (53.5%)
	Mean 59.63 ± 10.79	Turkoman (1%)

**Table 2:** Carriage rate of pathogenic and/or likely pathogenic variants found in genes relevant to Mendelian diseases in 100 participants.

GENE	Carriage Rate (%)	Related Disease	GENE	Carriage Rate (%)	Related Disease
*CYP21A2*	14.7	Adrenal hyperplasia due to 21-hydroxylase deficiency	*C12orf65*	1.47	Combined oxidative phosphorylation deficiency 7 and spastic paraplegia 55
*HBB*	11.76	Thalassemia, β	*CCBE1*	1.47	Hennekam lymphangiectasia–lymphedema syndrome 1
*BTD*	10.29	Biotinidase deficiency	*CDK10*	1.47	Al Kaissi syndrome
*CFTR*	8.82	Cystic fibrosis	*CEP135*	1.47	Microcephaly 8
*RBM8A*	8.82	Thrombocytopenia-absent radius syndrome	*CHRNG*	1.47	Escobar syndrome and multiple pterygium syndrome, lethal type
*GAA*	5.88	Glycogen storage disease II	*MUTYH*	1.47	Adenomas, multiple colorectal
*GJB2*	4.41	Deafness	*CLCN1*	1.47	Myotonia congenita
*PAH*	4.41	Phenylketonuria	*COL18A1*	1.47	Knobloch syndrome, type 1
*ATP7B*	2.94	Wilson disease	*KIAA0586*	1.47	Joubert syndrome 23 and short-rib thoracic dysplasia 14 with polydactyly
*GALC*	2.94	Krabbe disease	*SLC25A26*	1.47	Combined oxidative phosphorylation deficiency 28
*PYGM*	2.94	McArdle disease	*FANCE*	1.47	Fanconi anemia, complementation group E
*COL4A3*	2.94	Alport syndrome	*FTCD*	1.47	Glutamate formiminotransferase deficiency
*CYP11B2*	2.94	Hypoaldosteronism	*G6PD*	1.47	Hemolytic anemia, G6PD-deficient (favism)
*ECHS1*	2.94	Mitochondrial short-chain enoyl-CoA hydratase 1 deficiency	*GHR*	1.47	Laron dwarfism
*HBA1*	2.94	Thalassemia, α	*HBA2*	1.47	Thalassemia, α
*LAMA2*	2.94	Muscular dystrophy	*MFSD8*	1.47	Ceroid lipofuscinosis, neuronal, 7 and Macular dystrophy with central cone involvement
*OPHN1*	2.94	Intellectual developmental disorder, Billuart type	*MOCS2*	1.47	Molybdenum cofactor deficiency B
*POLR3A*	2.94	Wiedemann–Rautenstrauch syndrome and leukodystrophy	*PGAP3*	1.47	Hyperphosphatasia with impaired intellectual development syndrome 4
*MYH7*	1.47	Congenital myopathy 7B, myosin storage	*NPHS1*	1.47	Nephrotic syndrome, type 1
*IFIH1*	1.47	Immunodeficiency 95	*PKLR*	1.47	Pyruvate kinase deficiency
*EXPH5*	1.47	Epidermolysis bullosa simplex 4	*PYROXD1*	1.47	Myopathy, myofibrillar, 8
*POMT1*	1.47	Muscular dystrophy	*TNFRSF13B*	1.47	Immunodeficiency, common variable, 2
*ACADM*	1.47	Deficiency of acyl-CoA dehydrogenase, medium-chain	*ABCD1*	1.47	Adrenoleukodystrophy
*NAGS*	1.47	N-acetylglutamate synthase deficiency	*NPC1*	1.47	Niemann–Pick disease
*SUCLG1*	1.47	Mitochondrial DNA depletion syndrome 9	*PLOD2*	1.47	Bruck syndrome 2
*ASS1*	1.47	Citrullinemia	*DHTKD1*	1.47	α-aminoadipic and α-ketoadipic aciduria
*CYP27A1*	1.47	Cerebrotendinous xanthomatosis	*GALT*	1.47	Galactosemia
*AMT*	1.47	Glycine encephalopathy 2	*SBDS*	1.47	Shwachman–Diamond syndrome 1
*PCCA*	1.47	Propionicacidemia	*WDR35*	1.47	Cranioectodermal dysplasia 2 and Short-rib thoracic dysplasia 7 with or without polydactyly
*SEC23B*	1.47	Dyserythropoietic anemia, congenital, type II	*ABCC6*	1.47	Arterial calcification, generalized, of infancy, 2 and Pseudoxanthoma elasticum
*FBXL4*	1.47	Mitochondrial DNA depletion syndrome 13	*COLQ*	1.47	Myasthenic syndrome, congenital, 5
*CTSK*	1.47	Pycnodysostosis	*GCH1*	1.47	Dystonia, DOPA-responsive and hyperphenylalaninemia, BH4-deficient, B

**Table 3:** Pathogenic and/or likely pathogenic variants found in each gene in the study population.

Gene	Variants Observed in Northern Cyprus	Gene	Variants Observed in Northern Cyprus
*CYP21A2*	NM_000500.5:c.850A>G, p.(Met284Val), rs770199817	*ACADM*	NM_001286043.1:c.244dup, p.(Trp82Leufs*15)
	NM_000500.5:c.844G>T, p.(Val282Leu)	*NAGS*	NM_153006.2:c.1552G>A, p.(Ala518Thr), rs745511282
	NM_000500.5: c.293-13C>G	*SUCLG1*	NM_003849.3:c.823dup, p.(Ser275Phefs*38)
	NM_000500.5:c.1174G>A, p.(Ala392Thr), rs202242769	*ASS1*	NM_000050.4:c.1168G>A, p.(Gly390Arg), rs121908641
	NM_000500.5:c.1360C>T, p.(Pro454Ser), rs6445	*CYP27A1*	NM_000784.3:c.1184+1G>A, rs587778777
*HBB*	NM_000518.4: c.93-21G>A, rs35004220	*AMT*	NM_000481.3:c.992G>A, p.(Arg331Gln)
	NM_000518.4:c.118C>T, p.(Gln40*), rs11549407	*PCCA*	NM_000282.3:c.1495del, p.(Ile499Serfs*19)
	NM_000518.4:c.20A>T, p.(Glu7Val), rs334	*SEC23B*	NM_001172745.1:c.40C>T, p.(Arg14Trp), rs121918222
*BTD*	NM_001281723.2:c.1336G>C, p.(Asp446His), rs13078881	*FBXL4*	NM_001278716.1:c.616C>T, p.(Arg206*), rs964532159
	c.1489C>T, p.Pro497Ser, rs138818907	*CTSK*	NM_000396.3:c.721C>T, p.(Arg241*), rs74315303
	NM_000060:c.1330G>C, p.Asp444His, rs13078881	*C12orf65*	NM_001143905.2:c.248del, p.(Val83Glyfs*2), rs587776508
*CFTR*	NM_000492.3:c.3872A>G, p.(Gln1291Arg), rs397508621	*CCBE1*	NM_133459.3:c.521G>A, p.(Cys174Tyr)
	NM_000492.3:c.2249C>T, p.(Pro750Leu), rs140455771	*CDK10*	NM_052988.4: c.609-1G>A, rs767176610
	NM_000492.3:c.3205G>A, p.(Gly1069Arg), rs200321110	*CEP135*	NM_025009.4:c.2722C>T, p.(Arg908*), rs186530606
	c.2991G>C, p.Leu997Phe, rs213950	*CHRNG*	NM_005199.4:c.753_754del, p.(Val253Alafs*44), rs767503038
	NM_000492.3:c.3472C>T, p.(Arg1158*), rs79850223	*MUTYH*	NM_001128425.1:c.884C>T, p.(Pro295Leu), rs374950566
	c.2421A>G, p.Ile807Met, rs1800103	*CLCN1*	NM_000083.2: c.854G>A, p.(Gly285Glu), rs150885084
*RBM8A*	NM_005105.4:c.-21G>A, rs139428292	*COL18A1*	ENST00000359759.8:c.4768_4769del, p.(Leu1590Valfs*72), rs398122391
*GAA*	NM_000152.3:c.1194+45G>A, rs369080138	*KIAA0586*	NM_001244189.1:c.428del, p.(Arg143Lysfs*4), rs534542684
	NM_000152.3:c.-32-13T>G, rs386834236	*SLC25A26*	ENST00000354883.10:c.316C>T, p.(Arg106*), rs150756149
	NM_000152.3:c.1828G>A, p.(Ala610Thr), rs144731405	*FANCE*	NM_021922.2:c.355C>T, p.(Gln119*), rs121434505
*GJB2*	NM_004004.5:c.35del, p.(Gly12Valfs*2), rs80338939	*FTCD*	NM_001320412.1:c.990dup, p.(Pro331Alafs*2), rs398124234
	NM_004004.5:c.269T>C, p.(leu90Pro), rs80338945	*G6PD*	NM_000402.3:c.653C>T, p.(Ser218Phe), rs5030868
*PAH*	NM_000277.1:c.1066-11G>A, rs5030855	*GHR*	NM_001242399.2: c.739T>C, p.(Tyr247His), rs143814221
	NNM_000277.1:c.1139C>T, p.(Thr380Met)	*HBA2*	NM_000517.4:c.*92A>G, rs63750067
*ATP7B*	NM_000053.2:c.122A>G, p.(Asn41Ser), rs201738967	*MFSD8*	NM_152778.2:c.1361T>C, p.(Met454Thr), rs559155109
	NM_000053.2:c.3053C>T, p.(Ala1018Val), rs371840514	*MOCS2*	NM_004531.3:c.471_477delinsG, p.(Leu158_Lys159del)
*GALC*	NM_000153.3:c.956A>G, p.(Tyr319Cys), rs183105855	*PGAP3*	NM_033419.3:c.*559_*560inv
	NM_000153.3:c.1901T>C, p.(Leu634Ser), rs138577661	*NPHS1*	NM_004646.3:c.1379G>A, p.(Arg460Gln), rs386833880
*PYGM*	NM_005609.3:c.808C>T, p.(Arg270*), rs767739769	*PKLR*	NM_000298.5:c.1456C>T, p.(Arg486Trp)
*COL4A3*	NM_000091.3:c.4421T>C, p.(Leu1474Pro), rs200302125	*PYROXD1*	NM_024854.3:c.464A>G, p.(Asn155Ser), rs781565158
*CYP11B2*	NM_000498.3:c.788T>A, p.(Ile263Asn)	*TNFRSF13B*	NM_012452.2:c.204dup, p.(Leu69Thrfs*12), rs72553875
*ECHS1*	NM_004092.3:c.476A>G, p.(Gln159Arg)	*ABCD1*	NM_000033.3:c.1699C>T, p.(Gln567*), rs201114595
	NM_004092.3:c.538A>G, p.(Thr180Ala), rs557128093	*NPC1*	NM_000271.4:c.506A>T, p.(Asn169Ile)
*HBA1*	NM_000558.3: c.95+2_95+6del	*PLOD2*	NM_182943.2:c.1856G>A, p.(Arg619His), rs121434461
*LAMA2*	NM_000426.3: c.2451-2A>G, rs993196576	*DHTKD1*	NM_018706.6:c.2185G>A, p.(Gly729Arg), rs117225135
*OPHN1*	NM_002547.3:c.1484del, p.Lys495Argfs*9	*GALT*	NM_0.000155.3: c.940A>G, p.N314D, rs2070074
*POLR3A*	NM_007055.3:c.1771-7C>G, rs201314157	*SBDS*	NM_016038.2:c.258+2T>C, rs113993993
	NM_007055.3:c.1771-7C>G, rs201314157	*WDR35*	NM_001006657.1:c.1922T>G, p.(Leu641*), rs199952377
*MYH7*	NM_000257.2:c.2609G>A, p.(Arg870His), rs36211715	*ABCC6*	NM_001171.5:c.3421C>T, p.(Arg1141*), rs72653706
*IFIH1*	NM_022168.3:c.2465G>A, p.(Arg822Gln), rs376048533	*COLQ*	NM_005677.3:c.1082del, p.(Pro361Leufs*65), rs769982050
*EXPH5*	NM_015065.2:c.2004_2014del, p.(Thr670Cysfs*8)	*GCH1*	NM_000161.2:c.671A>G, p.(Lys224Arg), rs41298442
*POMT1*	NM_007171.3:c.598G>C, p.(Ala200Pro), rs119462982		

## Data Availability

The datasets generated for this study are available on request to the corresponding author.

## References

[1] Barbitoff Y.A., Skitchenko R.K., Poleshchuk O.I., Shikov A.E., Serebryakova E.A., Nasykhova Y.A., Polev D.E., Shuvalova A.R., Shcherbakova I.V., Fedyakov M.A. (2019). Whole-exome sequencing provides insights into monogenic disease prevalence in Northwest Russia. Mol. Genet. Genomic Med..

[2] Park K.-S. (2021). Analysis of Worldwide Carrier Frequency and Predicted Genetic Prevalence of Autosomal Recessive Congenital Hypothyroidism Based on a General Population Database. Genes.

[3] Parikh V.N., Ashley E.A. (2017). Next Generation Sequencing in Cardiovascular Disease: Present Clinical Applications and the Horizon of Precision Medicine. Circulation.

[4] Schrodi S.J., DeBarber A., He M., Ye Z., Peissig P., Van Wormer J.J., Haws R., Brilliant M.H., Steiner R.D. (2015). Prevalence estimation for monogenic autosomal recessive diseases using population-based genetic data. Hum. Genet..

[5] Zhang L., Yu L., Shu X., Ding J., Zhou J., Zhong C., Pan B., Guo W., Zhang C., Wang B. (2023). Whole exome sequencing reveal 83 novel Mendelian disorders carrier P/LP variants in Chinese adult patients. J. Hum. Genet..

[6] Quaio C.R.D.C., Chung C.H., Perazzio S.F., Dutra A.P., Moreira C.M., Filho G.M.N., Sacramento-Bobotis P.R., Penna M.G., de Souza R.R.F., Cintra V.P. (2021). Frequency of carriers for rare recessive Mendelian diseases in a Brazilian cohort of 320 patients. Am. J. Med Genet. Part C: Semin. Med Genet..

[7] Chau J.F.T., Yu M.H.C., Chui M.M.C., Yeung C.C.W., Kwok A.W.C., Zhuang X., Lee R., Fung J.L.F., Lee M., Mak C.C.Y. (2022). Comprehensive analysis of recessive carrier status using exome and genome sequencing data in 1543 Southern Chinese. npj Genom. Med..

[8] Hernandez-Nieto C., Alkon-Meadows T., Lee J., Cacchione T., Iyune-Cojab E., Garza-Galvan M., Luna-Rojas M., Copperman A.B., Sandler B. (2020). Expanded carrier screening for preconception reproductive risk assessment: Prevalence of carrier status in a Mexican population. Prenat. Diagn..

[9] Gambin T., Jhangiani S.N., Below J.E., Campbell I.M., Wiszniewski W., Muzny D.M., Staples J., Morrison A.C., Bainbridge M.N., Penney S. (2015). Secondary findings and carrier test frequencies in a large multiethnic sample. Genome Med..

[10] Nazareth S.B., Lazarin G.A., Goldberg J.D. (2015). Changing trends in carrier screening for genetic disease in the United States. Prenat. Diagn..

[11] Kim Y.-E., Ki C.-S., Jang M.-A. (2019). Challenges and Considerations in Sequence Variant Interpretation for Mendelian Disorders. Ann. Lab. Med..

[12] Richards S., Aziz N., Bale S., Bick D., Das S., Gastier-Foster J., Grody W.W., Hegde M., Lyon E., Spector E. (2015). Standards and guidelines for the interpretation of sequence variants: A joint consensus recommendation of the American College of Medical Genetics and Genomics and the Association for Molecular Pathology. Genet Med..

[13] Phedonos A., Shammas C., Skordis N., Kyriakides T., Neocleous V., Phylactou L. (2013). High carrier frequency of 21-hydroxylase deficiency in Cyprus. Clin. Genet..

[14] Gialluisi A., Menabò S., Baldazzi L., Casula L., Meloni A., Farci M.C., Mariotti S., Balestrino L., Ortolano R., Murru S. (2018). A genetic epidemiology study of congenital adrenal hyperplasia in Italy. Clin. Genet..

[15] Baumgartner-Parzer S.M., Nowotny P., Heinze G., Waldhäusl W., Vierhapper H. (2005). Carrier frequency of congenital adrenal hyperplasia (21-hydroxylase deficiency) in a middle European population. J. Clin. Endocrinol. Metab..

[16] Baş F., Kayserili H., Darendeliler F., Uyguner O., Günöz H., Apak M.Y., Atalar F., Bundak R., Wilson R.C., New M.I. (2009). CYP21A2 Gene Mutations in Congenital Adrenal Hyperplasia: Genotype-phenotype correlation in Turkish children. J. Clin. Res. Pediatr. Endocrinol..

[17] Nordenström A., Svensson J., Lajic S., Frisén L., Nordenskjöld A., Norrby C., Almqvist C., Falhammar H. (2019). Carriers of a Classic CYP21A2 Mutation Have Reduced Mortality: A Population-Based National Cohort Study. J. Clin. Endocrinol. Metab..

[18] Pignatelli D., Carvalho B.L., Palmeiro A., Barros A., Guerreiro S.G., Macut D. (2019). The Complexities in Genotyping of Congenital Adrenal Hyperplasia: 21-Hydroxylase Deficiency. Front. Endocrinol..

[19] Bozkurt G. (2007). Results from the North Cyprus Thalassemia prevention program. Hemoglobin.

[20] Arpacı A., Doğan S., Erdoğan H.F., El Ç., Cura S.E. (2021). Presentation of a new mutation in FMF and evaluating the frequency of distribution of the MEFV gene mutation in our region with clinical findings. Mol. Biol. Rep..

